# Apolipoprotein C3: form begets function

**DOI:** 10.1016/j.jlr.2023.100475

**Published:** 2023-11-14

**Authors:** Karin E. Bornfeldt

**Affiliations:** Division of Metabolism, Endocrinology and Nutrition, Department of Medicine, UW Medicine Diabetes Institute and Department of Laboratory Medicine and Pathology, University of Washington, Seattle, WA, USA

**Keywords:** Apolipoproteins, Atherosclerosis, Inflammation, LDL, Triglycerides, VLDL

## Abstract

Increased circulating levels of apolipoprotein C3 (APOC3) predict cardiovascular disease (CVD) risk in humans, and APOC3 promotes atherosclerosis in mouse models. APOC3’s mechanism of action is due in large part to its ability to slow the clearance of triglyceride-rich lipoproteins (TRLs) and their remnants when APOC3 is carried by these lipoproteins. However, different pools and forms of APOC3 exert distinct biological effects or associations with atherogenic processes. Thus, lipid-free APOC3 induces inflammasome activation in monocytes whereas lipid particle-bound APOC3 does not. APOC3-enriched LDL binds better to the vascular glycosaminoglycan biglycan than does LDL depleted of APOC3. Patterns of APOC3 glycoforms predict CVD risk differently. The function of APOC3 bound to HDL is largely unknown. There is still much to learn about the mechanisms of action of different forms and pools of APOC3 in atherosclerosis and CVD, and whether APOC3 inhibition would prevent CVD risk in patients on LDL-cholesterol lowering medications.

## APOC3 as a Marker and Mediator of Atherosclerotic Cardiovascular Disease

Although apolipoprotein C3 (APOC3) was discovered more than 50 years ago, it is now attracting a surge in interest as a potential new drug target for the prevention of cardiovascular disease (CVD). What are the reasons behind this excitement? First, genetic evidence based on loss-of-function variants of *APOC3* and Mendelian randomization studies strongly support a causative role for APOC3 in increasing plasma triglycerides and CVD risk (1, 2, 3, 4). In one large study, the lower CVD risk observed in *APOC3* loss-of-function heterozygotes was determined to be mediated by lower levels of remnant lipoprotein-cholesterol and not by low-density lipoprotein (LDL) cholesterol (4). Remnant lipoprotein particles can be defined as postlipolytic partially triglyceride-depleted particles derived from chylomicrons and very-low-density lipoprotein (VLDL) that are relatively enriched in cholesteryl esters and apolipoprotein E (APOE) (5). Because of the heterogenicity of remnant particle size and composition, no high-throughput assay for remnants is currently available for direct measurements in large clinical trials (5). In the above study (4), remnant cholesterol was estimated by subtracting LDL-cholesterol and HDL-cholesterol from total cholesterol, and in a subcohort was measured directly. LDL-cholesterol estimation by the Friedewald equation used in a majority of clinical measurements is problematic because it defines remnant cholesterol as triglycerides/5. Keeping this in mind, there was a strong correlation between estimated remnant-cholesterol and remnants measured directly by a biochemical assay (4). Several lines of research support the concept that remnant lipoproteins are more atherogenic than LDL (5, 6, 7, 8). Second, plasma levels of APOC3 predict CVD events in individuals with and without diabetes. Thus, a meta-analysis of 11 studies (close to 3,000 CVD cases) concluded that a 5 mg/dl increase in total APOC3 was associated with a 33% increase in CVD risk (9). Third, studies in animal models, including in a mouse model of type 1 diabetes, have shown that silencing of APOC3 prevents the formation of both early and advanced lesions of atherosclerosis characterized by necrotic core expansion (10, 11, 12), while mice transgenic for human APOC3 have 2- to 3-fold larger atherosclerotic lesions than controls (13). These findings in mice are consistent with an intravascular ultrasound study in humans, which demonstrated that the percentage of necrotic core volume in culprit lesions and the risk of a future CVD event were higher in participants with high plasma levels of APOC3 (>8.5 mg/dl) than in participants with APOC3 levels below that threshold (14). Together, these studies provide strong support for APOC3 as both a biomarker and causal mediator of atherosclerotic CVD. Several reviews have covered the topic of APOC3 as a potential promising therapeutic target for CVD prevention (6, 15, 16). Cardiovascular outcome trial data on the effectiveness of APOC3 inhibition are still needed to directly support this concept.

In addition, APOC3 appears to hold an important role as a CVD risk factor and mediator in people with diabetes, at least in the case of type 1 diabetes, in which plasma triglyceride levels are often within the optimal (<100 mg/dl) to borderline (100–150 mg/dl) range. Studies on 3 independent cohorts of participants with type 1 diabetes have shown that APOC3 positively predicts the risk of major CVD events independently of traditional CVD risk factors, such as age, sex, diabetes duration, blood glucose control, blood pressure, smoking, LDL-cholesterol, and HDL-cholesterol (10, 17, 18). In the FinnDiane study (18), the association of APOC3 with CVD risk remained statistically significant when adjusted for sex, diabetes duration, systolic blood pressure, blood glucose control, smoking status, LDL-cholesterol, the use of lipid-lowering medication and baseline diabetic kidney disease, but stratifying the analysis on participants with and without albuminuria revealed that APOC3 predicted CVD risk only in those with albuminuria, perhaps reflecting the worsened CVD risk factors, including triglycerides and calculated remnant cholesterol in participants with micro- or macroalbuminuria. Indeed, the association of APOC3 with CVD in participants with albuminuria lost statistical significance when calculated remnant cholesterol was added to the model, indicating that APOC3’s association with CVD was not independent of remnant cholesterol.

Whether plasma APOC3 also predicts CVD risk in type 2 diabetes is less clear, as different studies show discrepant results. For example, one study showed a positive association between both total APOC3 and APOC3 carried by APOB-lipoproteins with coronary heart disease in subjects with type 2 diabetes and median triglycerides in the normal range (19), whereas in another study, APOC3 levels associated negatively with CVD, despite a positive correlation with triglycerides (20). Baseline median plasma triglycerides were above the borderline range (>150 mg/dl) in both controls and CVD cases in the latter study. In another study in which plasma triglyceride levels were 150 mg/dl or lower, serum APOC3 levels positively predicted CVD in subjects with type 2 diabetes (21). In that study, the association between serum APOC3 and CVD risk remained significant when adjusted for age, sex, blood pressure, BMI, HbA1c, diabetes duration, smoking status, kidney function measured as estimated glomerular filtration rate (eGFR), insulin use, LDL-cholesterol, HDL-cholesterol, and triglycerides. The authors speculated that the different results might have been due to the APOC3 assay as performed in different laboratories.

Plasma APOC3 is positively associated with triglyceride levels, and APOC3 is therefore often elevated in states associated with hypertriglyceridemia and insulin resistance (22, 23). Indeed, the secretion rate of APOC3 is associated with elevated TRLs in subjects with type 2 diabetes (24). APOC3 levels might therefore increase in the pre-diabetic stage, before the development of overt type 2 diabetes.

In some clinical studies, the association of APOC3 with CVD was independent of plasma triglycerides but in others, it was not (9, 25). A dissociation of APOC3 levels from plasma triglyceride levels in some cases is also supported by mouse studies in which diabetes resulted in increased plasma APOC3, as compared with non-diabetic controls, when matched for plasma triglycerides (10).

Thus, APOC3’s mechanism of action does not appear to be explained solely by its effects on plasma triglycerides, and the mechanisms whereby APOC3 promotes atherosclerosis may go beyond its effects on circulating triglycerides and cholesterol.

## Different Pools and Forms of APOC3

**Fig. 1:**
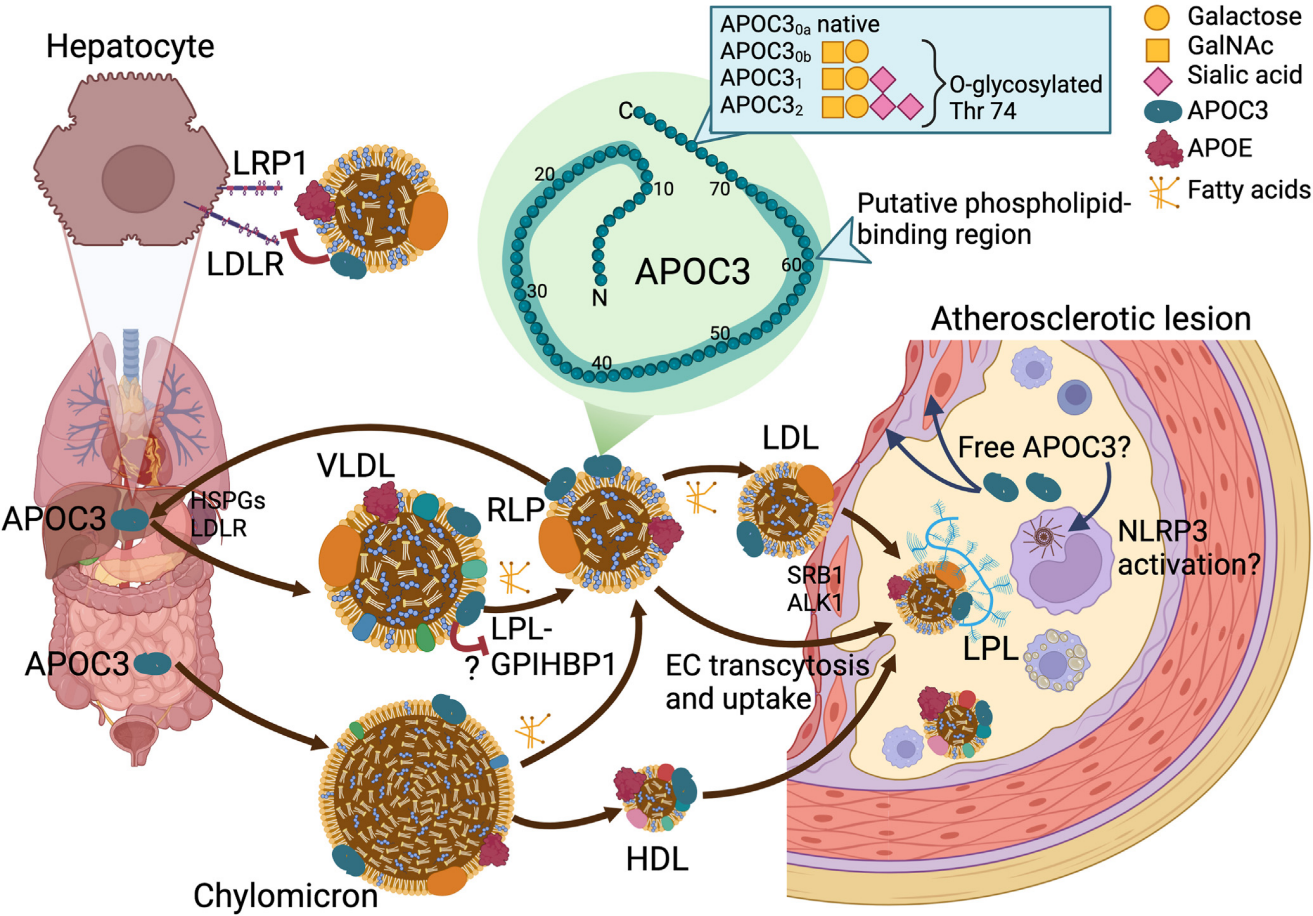
APOC3 and its potential mechanisms in atherosclerosis. APOC3 is produced primarily by hepatocytes, but also by enterocytes in the intestine. It circulates bound to triglyceride-rich lipoproteins (TRLs; VLDL and chylomicrons) and the remnant lipoproteins (RLPs) generated when lipoprotein lipase (LPL) hydrolyzes the triglyceride cargo of these lipoproteins for fatty acid uptake into tissues that use fatty acids as fuel or store fatty acids in the form of triglyceride-rich lipid droplets. Less APOC3 is associated with LDL. APOC3 is transferred from TRLs to HDL as part of TLR metabolism. APOC3 binds lipoprotein surface phospholipids through a putative phospholipid-binding region (highlighted by a green halo). APOC3 exists in several glycoforms in humans, characterized by different O-glycosylation patterns. These different proteoforms show discrepant associations with CVD and metabolic disease. APOC3 increases plasma triglyceride levels by inhibiting LPL activity and increases both triglycerides and cholesterol by interfering with the clearance of TRLs and their remnants by hepatic LDLR and LRP1. APOB-containing lipoproteins, which carry APOC3, enter lesions of atherosclerosis through endothelial transcytosis mediated by SRB1 and ALK1. Although virtually no lipid-free APOC3 exists in circulation, it is possible that APOC3 is liberated from lipoproteins in lesions, in which LPL is expressed by macrophages. Lipid-free APOC3 might induce NLRP3 inflammasome activation in newly recruited monocytes, and perhaps exert pro-atherogenic effects on other lesion cells, such as endothelial cells and smooth muscle cells. In addition, LDL and VLDL carrying APOC3 show enhanced binding to biglycan, a negatively charged glycosaminoglycan present in lesions, potentially causing increased vascular retention of atherogenic lipoproteins. Therefore, the mechanisms whereby APOC3 promotes atherosclerosis may go beyond its effects on circulating lipid levels. HSPGs, heparan sulfate proteoglycans. Created with BioRender.com.

APOC3 is a small (79 amino acids) apolipoprotein produced primarily by the liver and to a lesser extent by the intestine (Fig. 1). In circulation, APOC3 is bound to triglyceride-rich lipoproteins (TRLs), comprised of chylomicrons from the intestine and VLDL from the liver, their remnants, and to high-density lipoprotein (HDL). In addition, there is a small amount of APOC3 bound to LDL. APOC3 exchanges readily between these lipoprotein pools; APOC3 can bind to the surface phospholipids of all lipoproteins. The phospholipid-biding region of APOC3 generally is believed to be primarily located in the C-terminal half of the protein, which contains several amphipathic helices (26). Nuclear magnetic resonance (NMR) studies of APOC3 complexed to SDS micelles suggest that APOC3 wraps around the micelle surface using six amphipathic helices, some located closer to the N-terminus, curved and connected via semiflexible hinges (27) (Fig. 1). This allows APOC3 to bind to lipoproteins of very different sizes, such as chylomicrons (with a diameter up to 1200 nm) versus small HDL particles (7.8 nm diameter).

In this context, it is important to consider that different lipoprotein isolation methods can affect measured APOC3 levels in a particular lipoprotein pool. For example, a recent study showed similar levels of HDL-associated APOC3 when HDL was isolated by ultracentrifugation and dextran-sulfate precipitation, but lower APOC3 levels in immunoaffinity-isolated HDL (28). There is normally very little free APOC3 in circulation (29, 30).

Although a readily exchangeable apolipoprotein, APOC3 has different functions when carried by APOB-containing lipoproteins (TRLs, their remnant lipoprotein particles [RLPs] and LDL) versus the pool carried by HDL. When carried by APOB-lipoproteins, the best known functions of APOC3 are to inhibit lipoprotein lipase (LPL) action, thereby preventing optimal hydrolysis of the triglyceride cargo of those lipoproteins, and to interfere with hepatic clearance of APOB-lipoproteins. These functions are discussed in more detail below.

The function of APOC3 when bound to HDL is less well understood, but like several other apolipoproteins, APOC3 can (at least in vitro) bind to SRB1 (scavenger receptor class B member 1) (31), a multi-functional receptor with functions spanning cholesterol efflux from macrophages, to uptake of HDL cholesterol in the liver and other tissues (32) to endothelial transcytosis of HDL and LDL (33, 34). It is possible that HDL-associated APOC3 could block APOE-enhanced clearance of HDL via SRB1, that HDL populations that carry APOC3 have different functions than APOC3-free HDL (35), or that APOC3-loading of HDL or APOB-lipoproteins alters SRB1-mediated endothelial transcytosis. Several studies have shown that APOC3-containing HDL predicts CVD risk (36) and that it is associated with reduced insulin sensitivity more strongly than total plasma APOC3 (37).

In addition to the pools of APOC3 carried by different lipoproteins, APOC3 exists in several distinct glycoforms, glycosylated at Thr74 in human plasma. The most relevant glycoforms are the native APOC3_0a_ and three O-glycosylated forms: APOC3_0b_, with one galactose and one N-galactosamine (GalNAc); APOC3_1_ with one sialic acid added to the galactose and GalNAc and APOC3_2_, with two sialic acid residues added to the APOC3_0b_ form (38, 39) (Fig. 1). Of those, APOC3_1_ is most abundant in plasma and in all lipoproteins, although there is significant variability between individuals (38) and with race and ethnicity (39). The APOC3_1_ proteoform was more abundant in people with diabetes and obesity in the MESA (Multi-Ethnic Study of Atherosclerosis) cohort (39). Importantly, the ratio of APOC3_0b_/APOC3_1_ was negatively associated with CVD risk after full adjustment in models including plasma triglycerides, HDL-cholesterol, and total APOC3 levels (39). The role of the APOC3_0b_ proteoform is interesting because this form did not show a strong association with traditional CVD risk factors, including lipids. These results suggest that glycoforms of APOC3 might have functional differences, perhaps related to the binding of sialylated APOC3 to heparan sulfate proteoglycans (HSPG) (40). Mouse models are unsuitable for functional studies of APOC3 glycoforms because mice lack the relevant glycosylation enzymes, but one study in which human TRLs carrying different APOC3 glycoforms were injected into mice suggested that APOC3_2_ is preferentially cleared via an HSPG-dependent pathway and APOC3_1_ is cleared primarily via the LDL receptor (LDLR) and LDL receptor-related protein 1 (LRP1) (40). The authors suggested that the most negatively charged APOC3_2_ glycoform might have a higher affinity for smaller TRL particles that are preferentially cleared via the slower proteoglycan-mediated pathways (40).

A report that APOC3 can be post-translationally guanidinylated and that the mass-signal intensity of guanidinylated APOC3 associated with kidney disease and CVD outcomes requires further study (41). The authors speculated that guanidinylated APOC3 acts to promote inflammation in tissues, rather than to regulate plasma lipid levels. It is possible that APOC3 disassociates from lipoproteins and exerts pro-inflammatory actions in tissues, once carried there by APOB-lipoproteins or HDL, because as recently shown by Hsu *et al.* (29), only lipid-free APOC3 activates an inflammasome pathway in monocytes. This mechanism is discussed in more detail below.

Together, these findings show that distinct forms of APOC3 associate differently with CVD and support the proposal that different APOC3 forms or pools could have different biological functions.

## APOC3 Pools and Lipid Metabolism

APOC3 has two mechanisms of action in triglyceride and cholesterol metabolism, both related to slower clearance of TRLs (16, 42). Although APOC3 was found to increase VLDL production and APOB secretion in one study (43), later studies showed that endogenous APOC3 does not act through increasing VLDL production (44, 45, 46, 47). Early studies in APOC3 transgenic mice concluded that the hypertriglyceridemia in those mice is not due to increased hepatic TRL APOB production or decreased lipolysis by LPL but rather results primarily from decreased tissue uptake of TRLs from circulation (48).

Early in vitro studies indicated that APOC3 can directly inhibit LPL activity by binding to LPL (49) and suggested that this mechanism could explain the ability of APOC3 to increase plasma triglyceride levels by suppressing the margination of TRLs. However, later studies showed that APOC3 does not directly inhibit LPL’s catalytic activity, but instead prevents access of LPL to its triglyceride substrate. The ability of APOC3 to prevent triglyceride hydrolysis is enhanced when LPL is bound to glycosylphosphatidylinositol-anchored HDL-binding protein 1 (GPIHBP1), perhaps further limiting the ability of LPL to access TRL surface triglycerides (50) (Fig. 1). The ability of APOC3 to prevent LPL’s action could explain why in a very hypertriglyceridemic LDLR-deficient hamster model, APOC3-deficiency, despite lowering plasma triglycerides, resulted in increased atherosclerosis (51); it is possible that increased LPL-mediated hydrolysis of TRL triglycerides brought about by APOC3-deficiency might have resulted in increased atherogenic remnant formation.

Human *APOC3* loss-of-function carriers, compared with non-carriers, had lower fasting and postprandial serum triglycerides, higher levels of HDL-cholesterol, and lower levels of LDL-cholesterol (3), suggesting that APOC3’s effects extend beyond preventing triglyceride hydrolysis. Indeed, APOC3 silencing using an antisense oligonucleotide therapeutic (volanesorsen) was found to suppress not only plasma triglycerides but also non-HDL-cholesterol and VLDL-cholesterol in subjects with familial chylomicronemia syndrome, a rare genetic disorder characterized by reduced or absent LPL activity (52). There were also increases in LDL-cholesterol and total APOB and HDL-cholesterol in those subjects. Together, these findings strongly suggest that APOC3 prevents hepatic clearance of VLDL-cholesterol and remnant cholesterol and that the physiological effects of APOC3 on plasma triglycerides are not solely dependent on LPL. This conclusion is backed up by a mouse study, in which APOC3 silencing lowered both TRL triglyceride and cholesterol in LPL-deficient mice (44). Rather than inhibiting LPL action, APOC3 was found to prevent hepatic clearance of TRLs and their remnants by LDLR and LRP1. Moreover, a human study on *APOC3* loss-of-function carriers showed markedly reduced pool sizes of VLDL and intermediate-density lipoprotein (IDL) with no difference in LDL pool size, and an acceleration of both VLDL/IDL clearance by the liver and conversion to LDL (47). Although some of the effects of APOC3 loss-of-function on VLDL- and remnant-cholesterol might be explained by the preferential hepatic clearance of smaller TRLs (generated by lipolysis) versus larger TRLs, together these studies support a significant inhibitory effect of APOC3 on hepatic TRL clearance (Fig. 1).

Research on mouse models has also shown that APOC3 can prevent tissue LPL action in vivo, at least when APOE, a ligand for LDLR and LRP1, is absent (45, 53). There are also human data consistent with the effect of APOC3 on lipolysis. Thus, a study on heterozygous carries of a loss-of-function *APOC3* variant revealed higher fractional clearance rates for VLDL-triglycerides and VLDL-APOB100 and greater conversion rates of VLDL to LDL (46). This study concluded that reduced levels of APOC3 in plasma increase the lipolytic metabolism of VLDL rather than the hepatic uptake of VLDL particles or remnants.

The function of APOC3 when associated with HDL is much less clear, but several studies have shown positive associations of APOC3-containing HDL with CVD. In one large study, HDL that contained APOC3 was associated with a higher risk of incident CVD, whereas HDL deficient in APOC3 was associated with lower risk (36). It is not known if these associations were due to direct functional effects of APOC3 bound to HDL, if APOC3 marks a particular HDL subpopulation that might have detrimental properties, or if APOC3-HDL is an innocent bystander. Another possibility is that storage of clinical samples leads to increased accumulation of APOC3 in HDL, especially in subjects with high triglyceride levels (54). Perhaps HDL serves as an inert storage pool for APOC3 transferred from APOB-containing lipoproteins, or perhaps an APOC3-containing HDL pool has positive effects on CVD risk through indirect or direct unknown mechanisms.

Lipid-associated APOC3 is cleared from plasma through the liver. A mouse in which *Ndst1* (N-deacetylase and N-sulfotransferase 1; an enzyme that governs N-sulfation of heparan sulfate proteoglycans) was deleted selectively in hepatocytes showed accumulation of APOC3 in the TRL fraction (44), suggesting that APOC3-containing lipoproteins are cleared through a heparan sulfate proteoglycan-dependent pathway, like TRLs (55). Deletion of LDLR on top of the hepatic NDST1-deficiency enhanced accumulation of both cholesterol- and TRLs compared with mice lacking only LDLR. Thus, hepatic heparan sulfate is a critical part of the mechanism of clearing both cholesterol-rich lipoprotein particles and triglyceride-containing lipoprotein particles, and this effect is accentuated in states in which LDL receptors are downregulated (55). In vitro studies suggested that APOCs, like APOE, when associated with TRLs are recycled by hepatocytes and that APOCs are re-secreted into the extracellular space where they bind to HDL, whereas APOB is degraded (56, 57). A slower clearance of TRLs and their remnants likely explains the increased APOC3 plasma levels in an LDLR-deficient mouse model of type 1 diabetes (58).

Lipid-free APOC3 is cleared by the kidney, as demonstrated by studies on the APOC3 variant APOC3 Ala43Thr, which is associated with reduced plasma triglycerides and APOC3, and protection against CVD (59). Mouse studies showed that the reduction in plasma APOC3 in mice carrying this missense variant was due to impaired binding of the variant to lipoproteins and accelerated renal catabolism of free APOC3 (59).

Despite the different pools of APOC3, kinetics studies have shown that, because of APOC3’s rapid exchange between TRLs and HDL, it is not possible to determine APOC3’s distinct fractional secretion and clearance rates in different lipoprotein pools, and it is unclear if lipoprotein-specific APOC3 kinetics is even a meaningful concept (60). Thus, an increased APOC3 fractional clearance rate could indicate increased clearance of APOC3-containing TRLs/remnants by the liver, increased clearance of APOC3-containing HDL, or increased removal of free APOC3 from plasma (60).

Together, these studies show important physiological roles for APOC3 associated with TRLs and RLPs in slowing the hepatic clearance of these lipoproteins and also hampering lipolysis of these particles, which indirectly slows hepatic clearance because clearance of large TRLs is dependent on the initial action of LPL (61). The function of APOC3 associated with HDL and LDL needs further research.

## APOC3 Forms and Inflammation

There are several emerging links between APOC3 and inflammation, some of which are associated with APOC3’s regulation of triglycerides and cholesterol and others that are not. Serum APOC3 levels can be elevated in states of inflammation, including the autoinflammatory conditions rheumatoid arthritis (62), systemic lupus erythematosus (63), and Guillain-Barré syndrome (64). In one study, patients with high APOC3 levels undergoing coronary angiography had significantly higher markers of systemic inflammation, such as high-sensitivity C-reactive protein (hsCRP) (65). In some of these studies, APOC3 was associated with disease scores independently of plasma lipids (63). Moreover, in a recent mouse study, deletion of the NLR family pyrin domain containing 3 (NLRP3) inflammasome in hematopoietic chimeras with diabetes resulted in reduced plasma levels of APOC3 without a significant drop in plasma triglycerides (66) and human studies show an interaction between plasma APOC3 levels and an *NLRP3* variant that causes increased NLRP3 activity (67). It is therefore possible that some types of inflammation result in elevated APOC3 levels.

However, APOC3 can also cause inflammation, at least the delipidated form of APOC3. Thus, in vitro, delipidated APOC3 induces an alternative NLRP3 inflammasome activation pathway in human monocytes by causing dimerization and activation of Toll-like receptor (TLR) 4 and TLR2 (65). This effect did not appear to be due to endotoxin contamination of the APOC3 preparations. In addition to the release of IL-1β as a result of inflammasome activation, delipidated APOC3 induces expression and secretion of other inflammatory and pro-coagulatory mediators in human monocytes, including TNF-α, IL-6, and tissue factor (65, 68). A subsequent study replicated and extended these findings of APOC3-induced inflammasome activation in human monocytes to mouse monocytes, but also demonstrated that APOC3 lost the ability to activate inflammasomes when bound to lipid particles (29). In plasma, virtually all APOC3 is bound to TRLs, RLPs and HDL (and some to LDL), but it is possible that lipoprotein-free APOC3 could be generated in tissues, such as in lesions of atherosclerosis (Fig. 1). The physiological or pathological relevance of such a mechanism is uncertain, and further research is needed to address this possibility.

Another possibility is that APOC3-containing lipoproteins are more inflammatory than lipoproteins not containing APOC3, either due to the direct effects of APOC3 or to changes in the lipoprotein particle that makes it more inflammatory or atherogenic. For example, APOC3-containing LDL and VLDL bind better to the vascular proteoglycan biglycan than lipoproteins depleted of APOC3, and this effect is mediated by APOB rather than by direct APOC3 binding to the proteoglycan (69, 70). Moreover, APOC3-containing LDL shows differences in lipid composition and an increased susceptibility to hydrolysis and aggregation by sphingomyelinases (70). Other studies have found that APOC3-enriched TRLs increase cellular expression of inflammatory mediators through a TLR2/NFκB mechanism compared with the same concentration of TRLs with a low APOC3 content (71).

An important issue therefore is whether APOC3 promotes CVD risk in part through its stimulatory effects on inflammatory pathways. So far, evidence for this scenario is scant. For example, in the FinnDiane study on subjects with type 1 diabetes, systemic inflammation characterized by elevated levels of hsCRP had only minor effects on the association between APOC3 and CVD events (18), and an *APOC3* loss-of-function variant was associated with slightly increased hsCRP levels in the UK Biobank, rather than reduced levels, as one would expect if APOC3 increased hsCRP levels (29). Moreover, in mice deficient in hepatic LDLR and LRP1, APOC3 inhibition had little effect on plasma lipids and had no beneficial effects on advanced atherosclerosis, as opposed to mouse models in which APOC3 silencing lowers lipids (11), suggesting that enhanced inflammatory processes might not contribute in major ways to the pro-atherogenic effects of APOC3. However, inflammatory pathways in specific cell types were not evaluated in the latter study.

Rather than increasing systemic inflammation, APOC3-containing lipoproteins or free APOC3 might have a number of local effects in lesions of atherosclerosis. APOC3 presumably enters a lesion of atherosclerosis bound to APOB-containing lipoproteins (small VLDLs, RLPs or LDL) or bound to HDL. In lesions from diabetic mice, APOC3 co-localizes to a large extent with APOB in close proximity to macrophages (72). APOB-containing lipoproteins enter the lesion through endothelial transcytosis by binding to SRB1 and ALK1 (activin A receptor like type 1) on endothelial cells (33, 34). HDL also enters the lesion by endothelial transcytosis mediated by SRB1, and HDL can compete with LDL for SRB1-mediated transcytosis (73). One possibility is that APOC3-containing lipoproteins transcytose more effectively than APOC3-devoid lipoproteins (Fig. 1). Once in the lesion, it is conceivable that APOC3 is liberated from lipoproteins by the action of lipases, such as LPL expressed by lesion macrophages (74). Lipid-free APOC3 could then potentially induce NLRP3 inflammasome activation in newly recruited monocytes or perhaps in other lesion cell types (Fig. 1).

Together, these studies show that lipid-bound and lipid-free forms of APOC3 have differential effects on inflammatory processes. Addressing the possibility that APOC3 has local effects in lesions beyond regulation of circulating triglycerides and cholesterol requires new approaches, such as single-cell RNA-sequencing or spatial transcriptomics, lipidomics, and proteomics.

## Clinical Implications and Future Directions

Two strategies for lowering APOC3 have been developed and are in clinical trials or are already approved for pancreatitis prevention in patients with severe hypertriglyceridemia and familial chylomicronaemia syndrome. These include an APOC3 antisense oligonucleotide (volanesorsen), which is approved for use in Europe (75, 76) and more recently a liver-targeted antisense oligonucleotide (olezarsen) (77, 78), and a small interfering RNA (ARO-APOC3) (79). A monoclonal antibody that reduces APOC3 plasma levels by a mechanism that appears to involve increasing splenic uptake of the APOC3-antibody complex (59) is no longer in clinical trials.

APOC3 inhibition has received much interest as a potential CVD treatment approach for adults with and without diabetes with mild-to-moderate hypertriglyceridemia and increased CVD risk. Cardiovascular outcome trials that include subjects with and without diabetes would be the next important step (80). Including participants with diabetes would be important also because there are inconclusive indications that APOC3 inhibition could improve insulin sensitivity (42). However, there are several important considerations. For example, participants in the futile PROMINENT trial had a significant reduction in plasma APOC3 levels (81). This cardiovascular outcome trial on triglyceride-lowering by the fibrate drug pemafibrate showed that a 28% reduction in plasma APOC3 levels and a 26% reduction in VLDL-cholesterol and remnant-cholesterol is not sufficient for CVD prevention (81). The futility of this trial could be due to the lack of reduction in total plasma APOB levels, given that both LDL and TRLs and their remnants contribute to CVD risk cumulatively. The pemafibrate-mediated reductions in TRL remnants were accompanied by increases in plasma LDL-cholesterol and APOB levels, probably because the conversion of VLDL to LDL was not accompanied by increased hepatic removal of LDL, with no overall change in non-HDL-cholesterol or total cholesterol levels (81). It is most likely important to lower plasma LDL levels in concert with APOC3 inhibition and to optimize LDL-lowering drugs.

A clinical trial on triglyceride-lowering in subjects with moderate hypertriglyceridemia (200–500 mg/dl) by olezarsen showed much more marked reductions in APOC3 (74% for the highest dose of olezarsen) and a 58% reduction in VLDL-cholesterol, and olezarsen also modestly lowered plasma APOB levels by 10% (82). Importantly, a majority of the subjects in this trial had type 2 diabetes. Clinical trial design for a cardiovascular outcome trial on APOC3 inhibition will therefore be critical. Important considerations may be to utilize elevated levels of APOB, non-HDL-cholesterol and remnant-cholesterol measurements, rather than triglycerides, for inclusion, to enroll patients with adequate background LDL-lowering therapy, targeting LDL-cholesterol of 70 mg/dl or below, and to include a large number of subjects with diabetes as they might be likely to see a relatively greater CVD risk reduction (83). Another important consideration is the race and ethnicity of the participants. APOC3 levels have been shown to be highest in people of Hispanic origin and lowest in African Americans (84) and the low APOC3 appears to contribute to the low VLDL triglyceride levels in African American women (85). APOC3 inhibition should also be evaluated in cohorts including Asian Indians because *APOC3* loss-of-function variants appear to be less effective in lipid-lowering in Asian Indians than in people of European descent (86).

A cardiovascular outcome study on APOC3 lowering considering these criteria would provide a major advance in our understanding of APOC3 as a mediator of CVD risk in participants with and without diabetes.

## Supplemental data

This article contains supplemental data.

## Conflict of interest

The authors declare the following financial interests/personal relationships which may be considered as potential competing interests: K. E. B. serves on the scientific advisory board of Esperion Therapeutics, Inc.

## Figures and Tables

**Karin Bornfeldt PhD JLR AE:**
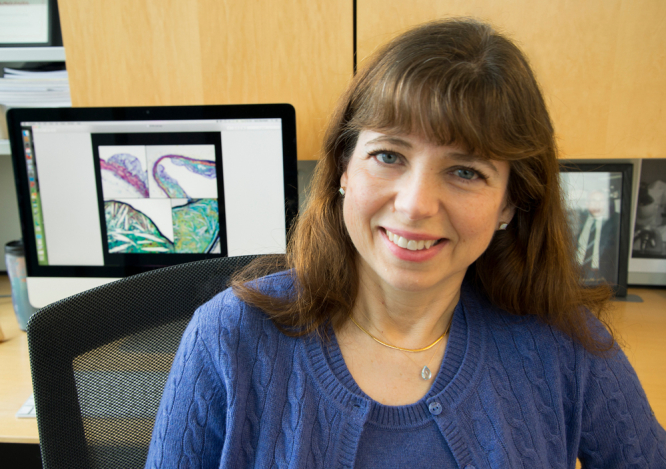


## Supplementary Data

Spotlight on JLR associate editor
