# Supplementary material for: Apolipoprotein C3: form begets function
Source: J Lipid Res. 2023 Nov 14;65(1):100475. doi: 10.1016/j.jlr.2023.100475 (PMC10805671; doi:10.1016/j.jlr.2023.100475)
Supplement: Spotlight on JLR associate editor [file mmc1.docx]

**Spotlight on JLR Associate Editor**

Karin Bornfeldt holds the Edwin L. Bierman Professorship in the Department of Medicine, Division of Metabolism, Endocrinology and Nutrition and is also Professor of Laboratory Medicine and Pathology at the University of Washington in Seattle. She was appointed as JLR Associate Editor in 2019.

• **What are your main research interests?** My research has been devoted to understanding how diabetes leads to an increased cardiovascular disease risk since I was a graduate student. We now analyze human samples to search for cardiovascular risk factors in people with diabetes and use mouse models of diabetes to provide insights into causality and mechanisms. Recently, we have been very interested in APOC3 as a potential mediator of residual cardiovascular disease risk in people with type 1 and type 2 diabetes and in its mechanisms of action.

• **How did you become interested in science?** I have been interested in science as long as I can remember. My parents instilled in me a curiosity for how nature works and an early awe in discovering. Many teachers and mentors along the way, as well as collaboration with a large number of exceptional colleagues and trainees have continued to inspire me throughout the years.

• **What was your pathway to becoming a JLR Associate Editor?** My pathway to becoming a JLR Associate Editor started by being asked to review manuscripts for JLR, then after some time being invited to serve on the Editorial Board. Returning constructive reviews in a timely manner was probably a key factor. After a few years, I was invited to become an Associate Editor. I believe peer-review is a very important part of our scientific community. I hope that by serving as Associate Editor for JLR, I can contribute to the publication of important and rigorous science focusing on lipids.

• **Who has had the most influence on your career?** My interest in becoming a scientist in the medical field was cemented through many inspiring mentors throughout my school years, graduate school and as a postdoc. My postdoc mentors Russell Ross and Edwin Krebs at the University of Washington had a tremendously positive influence on me and my career.

• **What do consider are the most important characteristics of successful trainees?** Passionate curiosity combined with keen observation skills. An ability to learn from and embrace situations that can feel like failures at the time; to grow as a person and scientist as a result. Science is becoming more collaborative, so an ability to collaborate with others, learn and exchange ideas with scientists from different backgrounds are additional key factors.

• **What advice do you have for writing successful funding applications?** The Specific Aims page is the most important part of a grant application. Successful applications explain the problem in simple language - why it is an important problem and how solving it will advance the field. After reading a well-constructed specific aims page, the reviewer should be excited about the research and wanting to know the answers. Beyond the Specific Aims page, scientific rigor is key.

• **What tips do you have for managing work-life balance?** I try to compartmentalize as much as possible, and to think carefully about priorities so that I can accept the most important tasks and have sufficient time to complete them carefully. I also rely on my very supportive family.

• **What do you enjoy doing in your spare time?** I love spending time in nature, still curious about how everything is connected, and being awed by the beauty of it all.
